# Clinical Exome Sequencing in Pediatric Patients

**DOI:** 10.7759/cureus.80330

**Published:** 2025-03-10

**Authors:** Orhan Görükmez, Özlem Görükmez, Ali Topak, Hanife Ayşegül Arsoy

**Affiliations:** 1 Medical Genetics, Bursa Yüksek İhtisas Training and Research Hospital, Bursa, TUR; 2 Medical Genetics, Bursa State Hospital, Bursa, TUR; 3 Pediatric Gastroenterology, Bursa State Hospital, Bursa, TUR

**Keywords:** clinical exome sequence, copy number of variants (cnv), next generation sequencing (ngs), novel mutation, orphan diseases

## Abstract

Introduction: The development of genomic sequencing techniques has led to the effective diagnosis of genetic diseases. In this study, clinical exome sequencing (CES) results applied to genetic disorders are reported.

Methods: The CES results of pediatric patients with different system involvements and whose complaints were thought to be of genetic origin were evaluated retrospectively.

Results: Significant variants associated with complaints were detected in 41 (60%) of 68 patients. Copy number variations were detected in two patients, and single nucleotide variants (SNVs) were detected in the other 39 patients. A total of 46 SNVs were detected in these 39 patients. Sixteen of the detected SNVs were previously reported in the literature, but 30 were novel.

Conclusions: This study shows that CES can provide a high diagnosis rate (60%) in childhood genetic diseases. Novel mutations (30) have contributed to the mutation profiles of genetic disorders.

## Introduction

It is assumed that around 300 million people worldwide have an orphan disease, the vast majority of which is of genetic origin [1]. With the increasing applicability of genomic sequencing technology, the molecular basis of more than 7100 genetic diseases has been identified in the Online Mendelian Inheritance in Man (OMIM) database [2]. Gene panels, clinical exome sequencing, and whole exome and genome sequencing are among the next-generation sequencing (NGS) techniques that are routinely applied today [3]. Approximately 85% of the variants that cause diseases are found in exons and splicing regions, and exome tests that enable sequencing of these regions have gained importance. Clinical exome sequencing (CES), designed to sequence exons and splice regions of genes with known phenotypic effects, is frequently used as a routine diagnostic test [4]. In this study, we aimed to present the results of the CES test in pediatric patients from 67 different families together with their clinical findings.

## Materials and methods

This retrospective study examined clinical findings and molecular genetic results of pediatric patients who presented to the Department of Medical Genetics at Bursa Yüksek İhtisas Training and Research Hospital, Bursa, between 2019 and 2022. The data were meticulously retrieved and curated from archived clinical and molecular records. Ethical approval for the study was obtained from the Ethics Committee of Bursa Yüksek İhtisas Training and Research Hospital (Approval No: 2011-KAEK-25 2022/11-07). The findings presented in this study were systematically derived from molecular analyses and bioinformatics evaluations, as described below in detail.

DNA samples were isolated from the peripheral blood of patients using a QIAamp DNA Mini Kit (Qiagen, Hilden, Germany). A total of 4493 genes were amplified from patient DNA samples using a CES kit (Clinical Exome Solution v2, Sophia Genetics SA., Saint Sulpice, Switzerland) on the NGS platform (NextSeq 500 system, Illumina, San Diego, California, USA), per the manufacturers’ instructions. The Sophia Data Driven Medicine (DDM^TM)^ platform (Sophia Genetics SA., Saint Sulpice, Switzerland) was used for bioinformatics analysis. All analyses of single nucleotide variants (SNVs), insertions and deletions (indels), and copy number variations (CNVs) were performed using this platform. In particular, variants with a minor allele frequency (MAF) <1%, associated with the clinical findings of the patients, were considered. Capillary electrophoresis was used to confirm variants that were considered significant and for segregation analysis. Primers for the variant-associated target sites were designed using Primer 3 (https://primer3.org/). A polymerase chain reaction (PCR) was performed using custom primers and H Taq polymerase (Zeydanlı, Ankara, Turkey) on a thermal cycler (Applied Biosystems^TM^ Veriti^TM^, Thermo Fisher Scientific, USA). Purification of the amplified products was carried out using a Zymo Research Sequencing Clean-up Kit (Epigenetic Companies, Irvine, CA, USA). Cycle sequencing was conducted using the BigDye Terminator v3.1 Cycle Sequencing Kit (Applied Biosystems®, Thermo Fisher Scientific, USA) on a 3500 Genetic Analyzer (Applied Biosystems®, Thermo Fisher Scientific, USA). The sequenced regions were analyzed using CLC Main Workbench v8.1 software (Qiagen).

Confirmation was obtained by fluorescence in situ hybridization (FISH) for the patient with heterozygous deletion of consecutive genes in the 5p subtelomere region in CNV analysis. A peripheral blood sample was cultured in RPMI-1640 medium supplemented with fetal bovine serum (FBS) and phytohemagglutinin (PHA). To obtain metaphase chromosomes, colcemid (0.1 µg/mL) was applied, followed by hypotonic treatment with 0.075 M KCl (37°C, 20 min). The cells were then fixed with a methanol:acetic acid solution (3:1). Genetic alterations associated with cri du chat syndrome were investigated using a specific fluorescent probe (Vysis ToTelVysion Multi-Color FISH Probe, Abbott Molecular, Des Plaines, IL, USA). Preparations were denatured at 75°C for two minutes and hybridized overnight at 37°C on a Leica ThermoBrite device (Leica Biosystems, Germany). Post-hybridization washes were performed using 0.4× saline-sodium citrate buffer (SSC)/0.3% nonidet P-40 (NP-40) (72°C), followed by a final wash with 2× SSC/0.1% NP-40 at room temperature. DAPI or 4',6-diamidino-2-phenylindole staining was applied to visualize nuclear structures. Fluorescent signals were analyzed using a fluorescence microscope (Allegro Plus, BioView, Rehovot, Israel), and at least 20 metaphases were assessed for the presence of 5p deletion.

Information was obtained on whether the variants were reported in the literature using the Human Gene Mutation Database (HGMD) [5]. Variants not reported in the literature at the time of this study were considered novel. The frequency of novel variants in the population was determined (gnomAD) [6] and categorized according to the American College of Medical Genetics and Genomics (ACMG) criteria [7]. Disease-related syndromes were determined in the OMIM database by evaluating the symptoms, family histories, examination findings, and detected variants.

## Results

This study was conducted on pediatric patients whose anamnesis, clinical complaints, and physical examination findings indicated a potential genetic basis for their condition. All data utilized in the study were meticulously compiled retrospectively from archived clinical and molecular records to ensure a comprehensive and systematic evaluation. Of the 68 patients included in the study, 39 were male, and 29 were female. Variants associated with clinical findings were detected in 41 patients: CNV in two patients and 46 SNVs in 39 patients (Table 1). One patient with SNV also had a mitochondrial DNA (mtDNA) mutation. In addition, an incidental variant was detected in another patient with an SNV. No variants that could cause disease were detected in the remaining 27 patients. A total of 46 different SNVs were detected, including the incidental one. Of the SNVs, 16 were previously reported in the literature, and 30 were novel. Novel variants were classified according to ACMG criteria; it was seen that 19 of them were of uncertain significance (UCS), nine of them were likely pathogenic (LP), and two of them were pathogenic (P). In addition, 25 of the SNVs were missense, eight were nonsense, eight were frameshift, three were splicing, and two were in-frame types. One of the CNVs was a partial homozygous deletion in intron 12 of the *DPYD *gene. The other mutation was a heterozygous deletion of more than one gene localized in the 5p region. Of all the variations, seven were de novo, and 40 were inherited from at least one parent. Segregation analysis could not be performed for these two variants.

**Table 1:** List of variants R/N: reported/novel; gnomAD: Genome Aggregation Database (https://gnomad.broadinstitute.org/) [6]; ACMG: American College of Medical Genetics and Genomics guideline (https://www.acmg.net/) [7]; P: pathogenic; LP: likely pathogenic; UCS: uncertain significance

Gene	Variant	Zygosity	Type	R/N	gnomAD	ACMG
ARSA NM_000487	c.827C>T (p.Thr276Met)	Heterozygous	Missense	CM930042		
	c.1393A>G (p.Lys465Glu)	Heterozygous	Missense	N	0	UCS
FANCA NM_000135	c.275C>A (p.Ser92*)	Heterozygous	Nonsense	CM080355		
	c.2738A>C (p.His913Pro)	Heterozygous	Missense	CM050606		
CHRNA2 NM_000742	c.1506G>C (p.Arg502Ser)	Heterozygous	Missense	N	0	UCS
KLF11 NM_001177716	c.1296_1297del (p.Lys435Glufs*8)	Heterozygous	Frameshift	N	0.0000159	UCS
GALNT12 NM_024642	c.1281_1296del (p.Trp427Cysfs*23)	Heterozygous	Frameshift	N	0.00000657	LP
TCOF1 NM_000356	c.38del (p.Leu13Argfs*18)	Heterozygous	Frameshift	N	0	LP
P53 NM_000546	c.733G>A (p.Gly254Ser)	Heterozygous	Missense	CM920674		
ARFGEF2 NM_006420	c.5063+2dupT	Homozygous	Splicing	N	0	UCS
NLRP1 NM_001033053	c.209C>A (p.Ala70Asp)	Heterozygous	Missense	N	0	UCS
	c.3338G>A (p.Arg1113His)	Heterozygous	Missense	N	0.00000797	UCS
MVK NM_000431	c.1129G>A (p.Val377Ile)	Homozygous	Missense	CM990890		
SCN3A NM_001081676	c.3220G>C (p.Glu1074Gln)	Heterozygous	Missense	N	0	UCS
ABHD5 NM_016006	c.594dupC (p.Arg199Glnfs*11)	Homozygous	Frameshift	CI013354		
TMEM5 NM_014254	c.1052delC (p.Pro351Leufs*6)	Homozygous	Frameshift	N	0	LP
RPE65 NM_000329	c.637C>T (p.Gln213*)	Homozygous	Nonsense	N	0	P
FAS NM_000043	c.854A>T (p.His285Leu)	Heterozygous	Missense	N	0	UCS
TCN2 NM_000355	c.244C>T (p.Gln82*)	Homozygous	Nonsense	N	0	LP
AIPL1 NM_001033054	c.683_688delinsA (p.Leu228Hisfs*115)	Homozygous	Frameshift	N	0	LP
MOCOS NM_017947	c.2368G>T (p.Glu790*)	Homozygous	Nonsense	N	0	LP
GBE1 NM_000158	c.1009T>G (p.Phe337Val)	Homozygous	Missense	N	0	UCS
ATP6V0A4 NM_020632	c.2419C>T (p.Arg807*)	Homozygous	Nonsense	N	0.0000239	UCS
COL1A1 NM_000088	c.2533G>A (p.Gly845Arg)	Heterozygous	Missense	CM090258		
GRIK1 NM_000830	c.332C>A (p.Ser111Tyr)	Homozygous	Missense	N	0	UCS
SH2D1A NM_001114937	c.213_224del (p.Val72_Arg75del)	Hemizygous	Inframe	N	0	LP
EIF2B4 NM_001034116	c.1409A>C (p.His470Pro)	Homozygous	Missense	N	0	UCS
ACAD9 NM_014049	c.797G>A (p.Arg266Gln)	Homozygous	Missense	CM108108		
HK1 NM_000188	c.1586T>C (p.Leu529Ser)	Homozygous	Missense	CM950628		
TRPM6 NM_001177310	c.151C>T (p.Arg51*)	Homozygous	Nonsense	CM025230		
WNK4 NM_001321299	c.1591_1593del (p.Ser532del)	Heterozygous	Inframe	N	0	UCS
CACNA1A NM_000068	c.6661_6662ins31 (p.His2221Profs*?)	Heterozygous	Frameshift	N	0	LP
SCN2A NM_001040142	c.4606A>G (p.Ser1536Gly)	Heterozygous	Missense	N	0	UCS
CDKL5 NM_001037343	c.730T>A (p.Phe244Ile)	Heterozygous	Missense	N	0	UCS
SLC5A2 NM_003041	c.655G>A (p.Ala219Thr)	Homozygous	Missense	CM061199		
ACTA1 NM_001100	c.282C>A (p.Asn94Lys)	Heterozygous	Missense	CM115391		
FANCL NM_001114636	c.974C>G (p.Pro325Arg)	Homozygous	Missense	N	0	UCS
SLC2A9 NM_001001290	c.162+1del	Homozygous	Splicing	N	0	P
XDH NM_000379	c.3710C>G (p.Pro1237Arg)	Homozygous	Missense	N	0.00000398	UCS
IKBKG NM_001099856	c.299C>T (p.Pro100Leu)	Hemizygous	Missense	N	0	UCS
INPP5E NM_019892	c.1697G>T (p.Gly566Val)	Homozygous	Missense	N	0	UCS
BBS7 NM_176824	c.712_715delAGAG (p.Arg238Glufs*59)	Homozygous	Frameshift	CD093973		
ASS1 NM_000050	c.970+5G>A	Heterozygous	Splicing	CS951350		
	c.1168G>A (p.Gly390Arg)	Heterozygous	Missense	CM900037		
CEP290 NM_025114	c.5557C>T (p.Gln1853*)	Heterozygous	Nonsense	N	0	LP
	c.5668G>T (p.Gly1890*)	Heterozygous	Nonsense	CM061683		

According to the Human Phenotype Ontology (HPO) filtering, connective tissue (n=1), craniofacial (n=1), gastrointestinal (n=2), genitourinary (n=1), hematological (n=13), immune (n=5), metabolic (n=6), mitochondrial (n=1), neurological (n=10), neurometabolic (n=3), neuromuscular (n=3), ophthalmic (n=1), and renal (n=7) diseases were detected. In addition, seven patients had multiple system disorders, five had a predisposition to cancer, and two had a complex phenotype. The incidental variant that we detected in one of our patients as having the potential to affect the endocrine system was not included in the HPO. In one of our patients, the incidental variant, which we thought was the cause of the maturity-onset diabetes of the young type 7 (MODY7), was not included in the HPO.

In the study group, there was a consanguineous marriage between the parents of 25 of our patients, and only two of these patients could not be diagnosed. Regardless of the consanguineous marriage, 14 of our patients had family members with the same molecular diagnosis or similar symptoms.

In this study, 44 different syndromes reported in the OMIM database were identified in our patients. A phenotype associated with the *GRIK1 *variant detected in one of our patients has not yet been reported in the OMIM database. We know that 5p deletion syndrome is not inherited, and most other syndromes show an autosomal-recessive inheritance pattern (26/44). Others were autosomal-dominant (11/44) and X-linked recessive (2/44) inheritance types. An inheritance pattern for *GRIK1 *defect, MODY7, colorectal cancer susceptibility to one was not specified in OMIM. Mitochondrial encephalomyopathy, lactic acidosis, and stroke-like episodes (MELAS) show a mitochondrial inheritance pattern.

## Discussion

This investigation centered on pediatric patients presenting with suspected genetic disorders who underwent NGS, integrating their clinical findings, familial histories, and physical examination data. The study synthesizes the demographic characteristics, clinical presentations, and molecular diagnostic outcomes of these patients. Specific cases are detailed to highlight unique findings and illustrate the diverse genetic etiologies observed in the cohort.

All variants detected in our patients were associated with different genes. Therefore, we did not have any two patients with the same diagnosis. There were individuals in a family with different diagnoses. Cases 53 and 54, with complaints of seizures, were siblings (Appendix 1). The male patient was born from a naturally conceived pregnancy, and the female patient was born from an in vitro fertilization (IVF) pregnancy. The variant (*SCN2A*, c.4606A>G, p․Ser1536Gly) detected in the male patient was inherited from his father, who had similar childhood complaints. The variant (*CDKL5*, c.730T>A, p.Phe244Ile) detected in the sister from the IVF pregnancy was de novo in a different gene. This situation raised the question, "Does the frequency of de novo mutations increase due to the IVF procedure?" There is no statistically significant difference in the incidence of de novo mutations between individuals born from medical-assisted reproductive pregnancies and those born from naturally conceived pregnancies [8]. A homozygous aryl hydrocarbon receptor interacting protein-like 1 (*AIPL1*) pathogenic variant was detected in case 35, who had congenital blindness. Since the patient also had findings suggestive of a neurometabolic disease, a whole mtDNA analysis was performed. We also detected the pathogenic variant m.5543T>C, which is compatible with MELAS. The coexistence of nuclear and mtDNA mutations has been reported rarely and mostly at the case level in the literature. We did not find the coexistence of these two diseases detected in our case [9-11].

**Figure 1:**
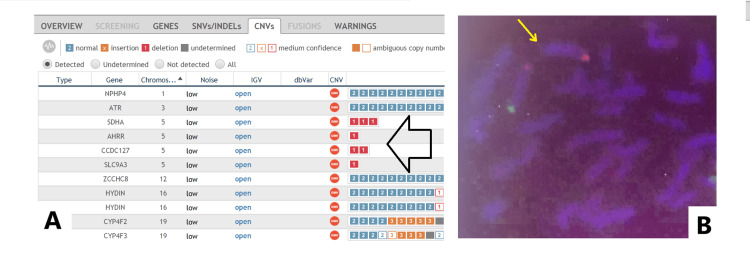
Detection of 5p15.33 deletion in the cri du chat syndrome patient by CNV analysis and FISH A: Copy number variation (CNV) analysis shows a heterozygous deletion in the 5p15.33 region, affecting the *SDHA*, *AHRR*, *CCDC127*, and *SLC9A3 *genes, which are indicated with a black arrow. B: Fluorescence in situ hybridization (FISH) image of a metaphase cell. The 5q telomeric region is marked with an orange signal, while the 5p telomeric region is labeled with a green signal. The deleted 5p telomeric region is indicated by a yellow arrow.

In our study, we diagnosed two patients using CNV analysis. Performing CNV analyses using exome sequencing has increased the diagnosis rate. It can provide an idea for small regions that cannot be detected using the microarray method [12]. We detected that the *CCDC127*, *SDHA*, *AHRR*, and *SLC9A3 *genes localized in the 5p15.33 region, respectively, were in a single copy number in an eight-year-old male patient numbered 29. We could not clarify the boundaries of the deletion because we could not apply MicroArray to the patient owing to technical limitations. We confirmed the deletion using a 5p subtelomeric FISH probe (Figure 1). The 5p deletion syndrome, also known as cri du chat syndrome, has a very heterogeneous phenotype due to differences in breakpoints [13]. Dysmorphic findings were indistinct, but the neurological symptoms were prominent. In the cranial magnetic resonance imaging of the patient, who was followed up for intellectual development and speech disorder, enlargement of the posterior horns of the lateral ventricles and periventricular hyperintensity were observed. The patient, who was followed up with a prediagnosis of hypoxic-ischemic encephalopathy until the age of eight years, was diagnosed with 5p deletion syndrome following CES. Another patient with CNV change was female (case 26). A section of intron 12 in the *DPYD *gene could not be sequenced. The fact that the CES kit covers this intronic region is advantageous. We detected a homozygous deletion of 380 bp by Sanger sequencing using custom primers. Dihydropyrimidine dehydrogenase deficiency caused by *DPYD *gene mutations can cause multiple organ disorders, mainly in the neurological system [14]. Our 15-month-old female patient had severe neurological problems and vision loss.

A homozygous missense variant (c.332C>A/p. Ser111Tyr) in the *GRIK1 *gene was detected in a four-month-old female infant (case 43) with severe neurometabolic and retinal problems. *GRIK1 *is among the genes that have not yet been definitively associated with a specific phenotype in the OMIM database. Chatterjee et al. associated *GRIK1 *gene alterations with attention deficit hyperactivity disorder [15], and Forero DA stated that it was a candidate gene for epilepsy [16]. This variant could not fully explain our patient's problems other than epilepsy; therefore, we suggested to her family that more comprehensive tests, such as whole genome sequencing (WGS), should be performed on the patient. Whether this change is an infrequent polymorphism or a disease factor will become clear as the number of cases increases.

In cases where secondary findings were detected in clinical exome studies, the method to be followed was determined according to ACMG criteria. It is recommended that the LP and P secondary variants of the *HNF1A *and *HNF1B *genes cause types 3 and 5, respectively, in the MODY group [17]. We detected a frameshift variant in *KLF11 *in addition to the *CHRNA2 *variant, which is the cause of the disease, in a six-year-old female patient (case 4) who underwent CES for febrile seizure complaints. *KLF11 *has been associated with MODY type 7 in OMIM, but its inheritance pattern is not specified (https://omim.org/). Notably, Laver et al. argued that the *KLF11 *gene is not associated with MODY in a study they conducted [18]. It is evident that this situation will become clear with the acceleration of functional studies and an increase in the number of cases. The *KLF11 *variant detected in our patient was inherited from the mother. Although the clinical significance of this variant is unknown according to the ACMG criteria, it has been reported that the mother was also diagnosed with diabetes mellitus at a young age. The patient was referred to the Pediatric Endocrinology Department.

The detection of the homozygous pathogenic *RPE65 *variant associated with Leber congenital amaurosis 2 (MIM:204100) in an 11-month-old male patient (case 30) is important in terms of treatment. Molecular diagnosis of diseases of genetic origin is undoubtedly of great importance in guiding disease treatment. Since 1990, when the first gene therapy was applied, studies have been conducted on gene therapy in many genetic diseases [19]. One of these diseases is Leber congenital amaurosis, which is caused by *RPE65 *mutations and has been approved for treatment [20]. Therefore, the patient was referred to the ophthalmology unit.

**Figure 2:**
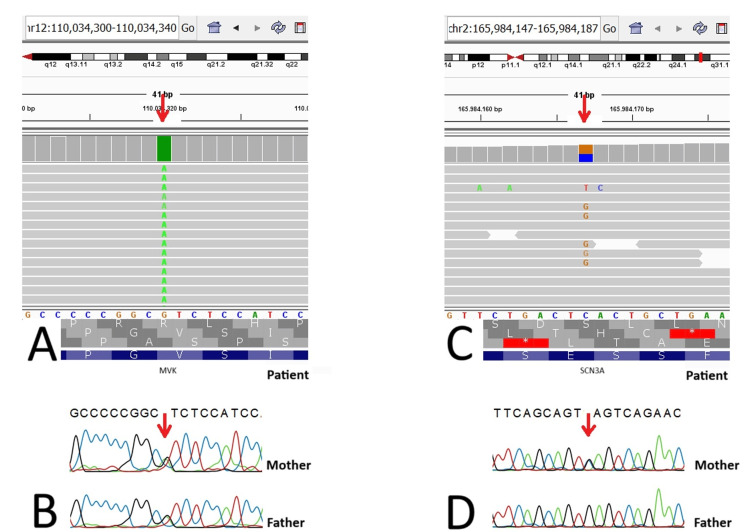
Genetic analysis of MVK and SCN3A variants in the patient and parents A: Integrated genomics viewer (IGV) visualization of the homozygous MVK; c.1129G>A; (p.Val377Ile) variant in the patient; B: Sanger sequencing chromatograms of the heterozygous MVK; c.1129G>A; (p.Val377Ile) variant in the parents; C: IGV track of the heterozygous SCN3A; c.3220G>C; (p.Glu1074Gln) variant in the patient, shown in the forward strand; D: Sanger sequencing results displaying the heterozygous SCN3A; c.3220G>C; (p.Glu1074Gln) variant in the mother and the wild-type sequence in the father, visualized in the reverse strand. The variant positions are indicated by red arrows.

More than one molecular pathology affecting the phenotype can be detected in comprehensive genetic tests such as CES [21]. Case 25 with the *MVK *pathogenic variant associated with hyper-IgD syndrome (MIM:260920) also carried the maternally inherited *SCN3A *variant (Figure 2). Although its clinical significance was uncertain, this variant was important to report since the mother had a history of childhood epilepsy.

In our study, which included the pediatric age group, the diagnosis rate was 60%. We attribute our relatively high diagnosis rate to our preference for CES over a panel of responsible genes in some cases with a specific phenotype but heterogeneous genetic etiology. Our CNV analysis may be another reason. However, when UCS variants are excluded, our diagnosis rate is 35% (24/68). CES has recently been used frequently in the diagnosis of genetic diseases because of its advantages, such as low cost, fast application, and easier analysis [22]. For these reasons, we preferred to apply CES to patients in our laboratory, which operates for diagnostic purposes. While the diagnostic range of CES is 50%-80% for diseases in the neonatal period, this rate decreases to 25%-50% for diseases presenting in adults or older people [22].

A notable limitation of this study is the inability to further investigate variants classified as uncertain significance (UCS) according to ACMG criteria. This limitation prevented the clarification of these variants, which might have provided additional insights into the molecular diagnoses. The other limitation of this study is that we were unable to perform MicroArray analysis to determine the exact breakpoints of the CNV detected in case 29 due to technical constraints.

We included 27 patients (~40%) who could not be diagnosed, and more extensive tests, such as whole-exome sequencing (WES) or WGS, were offered to these patients.

## Conclusions

Thirty novel variants associated with different syndromes were identified in the present study. Emphasis was placed on the clinical features and mutation profiles of genetic diseases, and complex phenotypes with different molecular etiologies must be considered. We believe that our study contributes to the literature in this respect.

## Appendices

Appendix 1 

**Table 2:** Demographic, clinical, and genetic findings of the patients R/N: reported/novel; gnomAD: Genome Aggregation Database (https://gnomad.broadinstitute.org/) [6]; ACMG: American College of Medical Genetics and Genomics guideline (https://www.acmg.net/) [7]; OMIM: Online Mendelian Inheritance in Man (https://www.omim.org/); IP: inheritance pattern; F: female; M: male; NB: newborn; y: year; m: month; P: pathogenic; LP: likely pathogenic; UCS: uncertain significance; AD: autosomal dominant; AR: autosomal recessive; XLR: X-linked recessive; U: unknown; MCA: multiple congenital abnormality; GIS: gastrointestinal system; GUS: genitourinary system; HUS: hemolytic uremic syndrome; HLH: hemophagocytic lymphohistiocytosis; MELAS: mitochondrial encephalomyopathy, lactic acidosis, and stroke-like episodes; IVF: in vitro fertilization; WMH: white matter hyperintensities

Case Number	Sex	Age	Clinical Findings	Gene	Transcript	Cdna	Amino Acid Change	Zygosity	Type	R/N	gnomAD	ACMG	Inherited From	OMIM	IP	Consanguinity	Family History	Disease Group
1	F	2y	Spastic tetraplegia, gait disturbance, cerebral white matter abnormalities	ARSA	NM_000487	c.827C>T	p.Thr276Met	Heterozygous	Missense	CM930042			Father	Metachromatic leukodystrophy (MIM: 250100)	AR	No	No	Neurometabolic
						c.1393A>G	p.Lys465Glu	Heterozygous	Missense	N	0	UCS	Mother					
2	M	5y	Gingival bleeding, thrombocytopenia	FANCA	NM_000135	c.275C>A	p.Ser92*	Heterozygous	Nonsense	CM080355			Mother	Fanconi anemia, complementation group A (MIM: 227650)	AR	No	No	Hematologic
						c.2738A>C	p.His913Pro	Heterozygous	Missense	CM050606			Father					
3	F	3y 6m	Macrothrombocytopenia	NORMAL												No	No	Hematologic
4	F	6y	Febrile seizures, normal psychomotor development	CHRNA2	NM_000742	c.1506G>C	p.Arg502Ser	Heterozygous	Missense	N	0	UCS	Father	Epilepsy, nocturnal frontal lobe, type 4 (MIM: 610353)	AD	No	No	Neurological
			Incidental	KLF11	NM_001177716	c.1296_1297del	p.Lys435Glufs*8	Heterozygous	Frameshift	N	0.0000159	UCS	Mother	Maturity-onset diabetes of the young, type VII (MIM: 610508)	?		No	Endocrine
5	M	12y 5m	Cafe-au-lait spots, multiple rectal polyps in him and his father,	NORMAL												No	Yes	Cancer
6	M	8y 6m	Intellectual developmental disorder, poor speech, history of operation due to pineal region tumor, mature teratoma, and bilateral inguinal hernia	NORMAL												No	No	MCA
7	F	3y	Bleeding, pancytopenia (aplastic anemia?)	NORMAL												No	No	Hematologic
8	F	11y 7m	Nausea, vomiting, hypertension, hemolytic anemia, thrombocytopenia, anuria (HUS?)	NORMAL												No	No	Renal
9	M	2y 7m	Cafe au lait spots (10 pieces), speech delay, familial history of cancer, microphthalmia, blindness, and optic atrophy on the left	GALNT12	NM_024642	c.1281_1296del	p.Trp427Cysfs*23	Heterozygous	Frameshift	N	0.00000657	LP	Mother	(Colorectal cancer, susceptibility to, 1) (MIM: 608812)	?	No	Yes	MCA
10	M	4y	Idiopathic thrombocytopenic purpura	NORMAL												No	No	Hematologic
11	M	7y 8m	Idiopathic thrombocytopenic purpura	NORMAL												No	No	Hematologic
12	F	NB	Dysmorphism	TCOF1	NM_000356	c.38del	p.Leu13Argfs*18	Heterozygous	Frameshift	N	0	LP	De novo	Treacher Collins syndrome 1 (MIM: 154500)	AD	No	No	Craniofacial
13	F	5m	HLH?	NORMAL												No	No	Hematologic
14	M	10y	Immunodeficiency	NORMAL												No	No	Immune
15	F	9y	History of ovarian germ cell tumor and acute myeloid leukemia	NORMAL												No	No	Cancer
16	M	11y	Immunodeficiency	NORMAL												Yes	No	Immune
17	M	3y	Recurrent bacterial infections, neutropenia	NORMAL												No	No	Hematologic
18	F	14y	Ganglioneuroblastoma, familial history of cancer	NORMAL												No	Yes	Cancer
19	M	4y 8m	Embryonal rhabdomyosarcoma and history of osteosarcoma in his sister	P53	NM_000546	c.733G>A	p.Gly254Ser	Heterozygous	Missense	CM920674			Father	Li-Fraumeni syndrome (MIM: 151623)	AD	No	Yes	Cancer
20	M	10y	Endobronchial carcinoid tumor	NORMAL												No	No	Cancer
21	M	18m	Jaundice, pruritus, intrahepatic cholestasis?	NORMAL												No	No	GIS
22	M	11m	Failure to thrive, microcephaly, hypotonia, seizures	ARFGEF2	NM_006420	c.5063+2dupT		Homozygous	Splicing	N	0	UCS	Parents	Periventricular heterotopia with microcephaly (MIM: 608097)	AR	Yes	No	Neurological
23	F	5y	Chronic diarrhea, eczema	NORMAL												No	No	GIS
24	M	17y	Fever attacks, xerosis, anemia, pyoderma gangrenosum, alopecia	NLRP1	NM_001033053	c.209C>A	p.Ala70Asp	Heterozygous	Missense	N	0	UCS	Mother	Autoinflammation with arthritis and dyskeratosis (MIM: 617388)	AR	No	No	Immune
						c.3338G>A	p.Arg1113His	Heterozygous	Missense	N	0.00000797	UCS	Father					
25	F	3y 5m	Vomiting, diarrhea, recurrent febrile attacks, seizures, delayed psychomotor development, microcephaly, cerebral atrophy	MVK	NM_000431	c.1129G>A	p.Val377Ile	Homozygous	Missense	CM990890			Parents	Hyper-IgD syndrome (MIM: 260920)	AR	Yes	No	Complex
				SCN3A	NM_001081676	c.3220G>C	p.Glu1074Gln	Heterozygous	Missense	N	0	UCS	Mother	Epilepsy, familial focal, with variable foci 4 (MIM: 617935)	AD		Yes	
26	F	15m	Microcephaly, epilepsy, hypertonia, motor retardation, nystagmus, bilateral congenital cataract, white matter lesions, cerebral atrophy, corpus callosum hypoplasia, multiple intracerebral microbleeds, optic atrophy	DPYD	NM_000110	c.1524+3011_1524+3390del		Homozygous	Deletion	N	0	UCS	U	Dihydropyrimidine dehydrogenase deficiency (MIM: 274270)	AR	Yes	No	Neurometabolic
27	M	2y	Congenital ichthyosiform erythroderma, hepatosplenomegaly, liver steatosis	ABHD5	NM_016006	c.594dupC	p.Arg199Glnfs*11	Homozygous	Frameshift	CI013354			Parents	Chanarin-Dorfman syndrome (MIM: 275630)	AR	Yes	No	MCA
28	M	9m	Congenital muscular dystrophy, lissencephaly	TMEM5	NM_014254	c.1052delC	p.Pro351Leufs*6	Homozygous	Frameshift	N	0	LP	Parents	Muscular dystrophy-dystroglycanopathy (congenital with brain and eye anomalies), type A, 10 (MIM: 615040)	AR	Yes	No	Neuromuscular
29	M	8y	Intellectual developmental disorder, speech delay, periventricular white matter abnormalities	5p15.33 (CCDC127, SDHA, AHRR, SLC9A3)				Heterozygous	Deletion				De novo	Chromosome 5p deletion syndrome (MIM: #123450)		Yes	No	Neurological
30	M	11m	Blindnes, nystagmus, strabismus	RPE65	NM_000329	c.637C>T	p.Gln213*	Homozygous	Nonsense	N	0	P	Parents	Leber congenital amaurosis 2 (MIM: 204100)	AR	Yes	No	Ophthalmic
31	M	2m	Hepatosplenomegaly, hemolytic anemia, thrombocytopenia, neutropenia	FAS	NM_000043	c.854A>T	p.His285Leu	Heterozygous	Missense	N	0	UCS	De novo	Autoimmune lymphoproliferative syndrome, type IA (MIM: 601859)	AD	No	No	Hematologic
32	M	12y 5m	Frequent infection history and fever, immunodeficiency (decreased IgG and IgM), alopecia areata, hepatomegaly, sparse eyebrows, thin and fragile nails	NORMAL												Yes	No	Immune
33	F	NB	Prematurity, hydrops, hepatomegaly, renal failure	NORMAL												No	No	MCA
34	M	8m	Diarrhea, vomiting, hypotonia, lethargy, and failure to thrive	TCN2	NM_000355	c.244C>T	p.Gln82*	Homozygous	Nonsense	N	0	LP	Parents	Transcobalamin II deficiency (MIM: 275350)	AR	Yes	Yes	Neurometabolic
35	F	11m	Pendular nystagmus, blindness from birth, hypotonia, lactic acidosis	AIPL1	NM_001033054	c.683_688delinsA	p.Leu228Hisfs*115	Homozygous	Frameshift	N	0	LP	Parents	Leber congenital amaurosis 4 (MIM: 604393)	AR	Yes	Yes	Complex
				MT-TW		m.5543T>C		Homoplasmic					Mother	MELAS Syndrome (MIM: #540000)	Mitochondrial		Yes	
36	F	10m	Petechiae, hepatomegaly, pancytopenia, hemophagocytosis (HLH?)	NORMAL												No	No	Hematologic
37	M	4y	Vomiting, xanthinuria	MOCOS	NM_017947	c.2368G>T	p.Glu790*	Homozygous	Nonsense	N	0	LP	Parents	Xanthinuria, type II (MIM: 603592)	AR	Yes	No	Metabolic
38	M	12y 6m	Dystonia	NORMAL												No	No	Neuromuscular
39	M	9m	Failure to thrive, hypotonia, hepatosplenomegaly	GBE1	NM_000158	c.1009T>G	p.Phe337Val	Homozygous	Missense	N	0	UCS	Parents	Glycogen storage disease IV (MIM: 232500)	AR	Yes	No	Metabolic
40	F	14y	Mullerian agenesis	NORMAL												No	No	GUS
41	F	3m	Growth deficiency, metabolic acidosis, hypercalciuria, hypomagnesemia, bilateral medullary nephrocalcinosis	ATP6V0A4	NM_020632	c.2419C>T	p.Arg807*	Homozygous	Nonsense	R			Parents	Distal renal tubular acidosis 3, with or without sensorineural hearing loss (MIM: 602722)	AR	Yes	Yes	Renal
42	F	NB	Numerous multiple fractures present at birth	COL1A1	NM_000088	c.2533G>A	p.Gly845Arg	Heterozygous	Missense	CM090258			De novo	Osteogenesis imperfecta, type II (MIM: 166210)	AD	No	No	Connective tissue
43	F	4m	Prematurity, microcephaly, retinopathy, epilepsy, hypotonia, hypoglycemia, lactic acidemia, ketonuria, elevated serum ammonia, metabolic encephalomyopathic crises, WMH	GRIK1	NM_000830	c.332C>A	p.Ser111Tyr	Homozygous	Missense	N	0	UCS	Parents	GRIK1 Deficiency (MIM: *138245)	AR?	Yes	Yes	Neurological
44	M	5y 2m	Hepatosplenomegaly, multiple cervical lymphadenopathy, anemia, thrombocytopenia, sepsis	SH2D1A	NM_001114937	c.213_224del	p.Val72_Arg75del	Hemizygous	Inframe	N	0	LP	Mother	Lymphoproliferative syndrome, X-linked, 1 (MIM: 308240)	XLR	No	No	Hematologic
45	F	7m	Hypotonia, seizures, developmental regression	EIF2B4	NM_001034116	c.1409A>C	p.His470Pro	Homozygous	Missense	N	0	UCS	Parents	Leukoencephalopathy with vanishing white matter (MIM: 603896)	AR	Yes	Yes	Neurological
46	M	1y	Hypotonia, encephalopathy, hypertrophic cardiomyopathy, lactic acidosis, thrombocytopenia	ACAD9	NM_014049	c.797G>A	p.Arg266Gln	Homozygous	Missense	CM108108			Parents	Mitochondrial complex I deficiency, nuclear type 20 (MIM: 611126)	AR	Yes	No	Mitochondrial
47	M	2y 2m	Difficulty walking, distal limb weakness, distal limb paralysis	HK1	NM_000188	c.1586T>C	p.Leu529Ser	Homozygous	Missense	CM950628			Parents	Neuropathy, hereditary motor and sensory, Russe type (MIM: 605285)	AR	Yes	No	Neurological
48	F	3m	Seizures, hypomagnesemia, hypocalcemia	TRPM6	NM_001177310	c.151C>T	p.Arg51*	Homozygous	Nonsense	CM025230			Parents	Hypomagnesemia 1, intestinal (MIM: 602014)	AR	Yes	Yes	Metabolic
49	M	2a	Microcephaly, global developmental delay	NORMAL												No	No	Neurological
50	M	3m	Hepatomegaly, fever, rash, pancytopenia (HLH?)	NORMAL												No	No	Hematologic
51	F	3m	Electrolyte imbalance, hyperkloremi, metabolic acidosis	WNK4	NM_001321299	c.1591_1593del	p.Ser532del	Heterozygous	Inframe	N	0	UCS	U	Pseudohypoaldosteronism, type IIB (MIM: 614491)	AD	No	No	Metabolic
52	F	4m	Epileptic encephalopathy, developmental delay, prematurity	CACNA1A	NM_000068	c.6661_6662ins31	p.His2221Profs*?	Heterozygous	Frameshift	N	0	LP	De novo	Developmental and epileptic encephalopathy 42 (MIM: 617106)	AD	No	No	Neurological
53	M	15m	Seizures, normal psychomotor development	SCN2A	NM_001040142	c.4606A>G	p.Ser1536Gly	Heterozygous	Missense	N	0	UCS	Father	Seizures, benign familial infantile, 3 (MIM; 607745)	AD	No	Yes	Neurological
54	F	18m	Seizures, EEG abnormalities, sleep difficulties, IVF	CDKL5	NM_001037343	c.730T>A	p.Phe244Ile	Heterozygous	Missense	N	0	UCS	De novo	Developmental and epileptic encephalopathy 2 (MIM: 300672)	AD	No	Yes	Neurological
55	M	1y	Renal glucosuria	SLC5A2	NM_003041	c.655G>A	p.Ala219Thr	Homozygous	Missense	CM061199			Parents	Renal glucosuria (MIM: 233100)	AR	Yes	No	Renal
56	F	5m	Polyhydramnios, decreased fetal movement, neonatal hypotonia, respiratory insufficiency due to muscle weakness	ACTA1	NM_001100	c.282C>A	p.Asn94Lys	Heterozygous	Missense	CM115391			De novo	Nemaline myopathy 3 (MIM: 161800)	AD	No	No	Neuromuscular
57	F	2y 1m	Abdominal pain, perinatal hydronephrosis, medullary nephrocalcinosis	NORMAL												No	No	Renal
58	F	15y	Pancytopenia, autoimmune lymphoproliferative disease?	NORMAL												No	No	Hematologic
59	M	9y	Hematochezia, fever, pancytopenia, bone marrow failure	FANCL	NM_001114636	c.974C>G	p.Pro325Arg	Homozygous	Missense	N	0	UCS	Parents	Fanconi anemia, complementation group L (MIM: 614083)	AR	Yes	No	Hematologic
60	M	15y	Hyperuricemic nephropathy	NORMAL												No	No	Renal
61	F	15m	Medullary nephrocalcinosis	NORMAL												No	No	Renal
62	F	13y	Low serum uric acid	SLC2A9	NM_001001290	c.162+1del		Homozygous	Splicing	N	0	P	Parents	Hypouricemia, renal, 2 (MIM: 612076)	AR	Yes	Yes	Renal
63	M	13y	Small kidneys, xanthinuria	XDH	NM_000379	c.3710C>G	p.Pro1237Arg	Homozygous	Missense	N	0.00000398	UCS	Parents	Xanthinuria, type I (MIM: 278300)	AR	Yes	No	Metabolic
64	M	17y	Recurrent infections, osteomyelitis	IKBKG	NM_001099856	c.299C>T	p.Pro100Leu	Hemizygous	Missense	N	0	UCS	Mother	Immunodeficiency 33 (MIM: 300636)	XLR	No	No	Immune
65	M	2y 6m	Dysmorphism, failure to thrive, delayed psychomotor development, history of neonatal breathing dysregulation, seizures, oculomotor apraxia, hypotonia, molar tooth sign seen on MRI, cerebellar vermis hypoplasia	INPP5E	NM_019892	c.1697G>T	p.Gly566Val	Homozygous	Missense	N	0	UCS	Parents	Joubert syndrome 1 (MIM: 213300)	AR	Yes	No	MCA
66	M	15y	Obesity, hypogenitalism, cryptorchidism on the right, history of surgery due to polydactyly on the hands and feet, retinitis pigmentosa	BBS7	NM_176824	c.712_715delAGAG	p.Arg238Glufs*59	Homozygous	Frameshift	CD093973			Parents	Bardet-Biedl syndrome 7 (MIM: 615984)	AR	Yes	No	MCA
67	M	NB	Coma, hyperammonemia	ASS1	NM_000050	c.970+5G>A		Heterozygous	Splicing	CS951350			Father	Citrullinemia (MIM: 215700)	AR	No	No	Metabolic
						c.1168G>A	p.Gly390Arg	Heterozygous	Missense	CM900037			Mother					
68	M	5y	Developmental delay and cognitive impairment, hypotonia, nystagmus, history of apnea in the neonatal period, renal cortical cysts, renal failure, cerebellar vermis aplasia, and molar tooth sign on MRI	CEP290	NM_025114	c.5557C>T	p.Gln1853*	Heterozygous	Nonsense	N	0	LP	Father	Joubert syndrome 5 (MIM: 610188)	AR	No	No	MCA
						c.5668G>T	p.Gly1890*	Heterozygous	Nonsense	CM061683			Mother					
